# Automating IoT Data Ingestion Enabling Visual Representation

**DOI:** 10.3390/s21248429

**Published:** 2021-12-17

**Authors:** Ala Arman, Pierfrancesco Bellini, Daniele Bologna, Paolo Nesi, Gianni Pantaleo, Michela Paolucci

**Affiliations:** Distributed Systems and Internet Technology Lab DISIT, University of Florence, 50139 Firenze, Italy; ala.arman@unifi.it (A.A.); pierfrancesco.bellini@unifi.it (P.B.); daniele.bologna@unifi.it (D.B.); gianni.pantaleo@unifi.it (G.P.); michela.paolucci@unifi.it (M.P.)

**Keywords:** IoT data ingestion, data warehouse, visual analytics

## Abstract

The Internet of things has produced several heterogeneous devices and data models for sensors/actuators, physical and virtual. Corresponding data must be aggregated and their models have to be put in relationships with the general knowledge to make them immediately usable by visual analytics tools, APIs, and other devices. In this paper, models and tools for data ingestion and regularization are presented to simplify and enable the automated visual representation of corresponding data. The addressed problems are related to the (i) regularization of the high heterogeneity of data that are available in the IoT devices (physical or virtual) and KPIs (key performance indicators), thus allowing such data in elements of hypercubes to be reported, and (ii) the possibility of providing final users with an index on views and data structures that can be directly exploited by graphical widgets of visual analytics tools, according to different operators. The solution analyzes the loaded data to extract and generate the IoT device model, as well as to create the instances of the device and generate eventual time series. The whole process allows data for visual analytics and dashboarding to be prepared in a few clicks. The proposed IoT device model is compliant with FIWARE NGSI and is supported by a formal definition of data characterization in terms of value type, value unit, and data type. The resulting data model has been enforced into the Snap4City dashboard wizard and tool, which is a GDPR-compliant multitenant architecture. The solution has been developed and validated by considering six different pilots in Europe for collecting big data to monitor and reason people flows and tourism with the aim of improving quality of service; it has been developed in the context of the HERIT-DATA Interreg project and on top of Snap4City infrastructure and tools. The model turned out to be capable of meeting all the requirements of HERIT-DATA, while some of the visual representation tools still need to be updated and furtherly developed to add a few features.

## 1. Introduction

Emerging smart cities bring forth the exploitation of big data analysis, including data collection, data ingestion, data processing, and data analytics, to produce hints about city conditions, evolution, and how to improve the quality of services and reduce costs. Smart cities are complex socio-technical systems composed of people (city users), stakeholders, organizations that may present competing objectives [1], infrastructure to provide connectivity, and process components (e.g., economy, mobility, environment, energy, and waste) [2]. In smart cities’ environments, different data sources (e.g., Internet of things, IoT, devices, points of interest, POIs, and geographical information systems) can be used to support different objectives (e.g., improving life quality, providing suggestions to city users and operators, and assisting decision makers by means of predictions, simulations, and plans) [3]. One of the major challenges in this context is dealing with the data ingestion process [1,4,5,6,7], addressing the import and pre-processing of complex multi-dimensional data (historical and real-time data with their metadata, which can be considered as static and real-time data, respectively). Acquired and derived data are stored to be further accessed and exploited for data analytics, dashboards, and visual analytics views [8]. In [9], a comparison of IPV4 and IPV6 has been presented to facilitate the transition to the latter one.

As a major research area in the big data age, in the context of smart cities, data ingestion and data warehouses have been made the subject of great attention by academic and industrial communities. The major challenge is the time needed to ingest new data models that may arise from several different devices and sources in the context of the IoT for smart cities and Industry 4.0. Such models may provide different data structures and meanings, and, as a result, distinct data dictionaries for single entities, etc. Additionally, despite the complexity, they should be ingested and understood to be used and visualized in a short time. Therefore, careful data ingestion, including regularization, indexing, and establishing relationships with other data entities in the domain, can save a huge amount of time in retrieving and accessing data for data analytics and visual rendering, which is typically requested in real-time, despite any large volume of data [10]. Data warehouse approaches can regularize data at ingestion time to simplify further exploitation [11]. On the contrary, other studies (e.g., [12]) have proposed a data lake architecture to reduce the data ingestion time, thus resulting in more complex processing during data access, data analytics, and rendering. The data lake approach may be suitable when a limited number of accesses to the same data are performed, but in a smart city data access is supposed to be massive via mobile apps and dashboards for city users.

### Overview

This paper presents models and tools for data ingestion and regularization, simplifying and enabling the automated visual representation of corresponding data. The state of the art of data warehouses includes solutions mainly based on ingestion processes which are not oriented to facilitate visual analytics. The addressed problems are related to the (i) regularization of the high heterogeneity of data that are available in the IoT devices (physical or virtual) and the KPIs (key performance indicators), as well as (ii) providing the final user with an index on views and data structures that can be directly exploited by graphical widgets of visual analytics tools. The proposed solution has addressed these problems by defining a formal model which allowed us to automate the activities of understanding data from data excel files, including time series and KPIs, which are processed to extract IoT device models, IoT devices, and their related time series data, including historical data.

The solution automatically identifies data models and generates IoT device models. Therefore, it can be very useful for registering IoT devices, creating data messages for IoT devices analyzing a single Excel File, understanding the data, and saving all messages automatically according to a well-formed conceptual model and format. It can be used for massive IoT device registration and historical data registration in a multitenant architecture. The proposed IoT device model is compliant with FIWARE NGSI V2 and supported by a formal definition of data characterization in terms of value type, value unit, and data type. The compliance with these aspects, geographical information and time series representation, allows heterogeneous data collected as hypercubes to be aggregated into a set of aligned hypercubes of cubes in terms of types, in time and space. The resulting model is coherent and used to generate data slices according to a number of operators to facilitate the activities of data selection, data visualization (via a wizard for the dashboard builder), and also to recover data via smart city APIs [13].

The solution has been developed in the context of the HERIT-DATA Interreg project [14]: in this framework six different pilots in Europe (Florence in Italy, Pont du Gard in France, Mostar in Bosnia–Herzegovina, Dubrovnik in Croatia, Patra in Greece, and Valencia in Spain) are involved in collecting complex big datasets as IoT devices and KPIs to exploit them for monitoring and reasoning people flows and tourism in general in order to improve service quality. With this aim, many heterogeneous data coming from several operators have been ingested and aggregated to provide representative dashboards that city operators can manage for business intelligence. The solution has been developed on top of Snap4City infrastructure and tools that can be accessed via the Snap4City.org framework [15]. The solution is GDPR (General Data Protection Regulation)-compliant [16] since the process respects data ownerships, performs any needed access delegations, and does not violate any mechanisms in place for the GDPR compliance of Snap4City.org.

The article is organized as follows: Section 2 presents the related work addressing the chain from data models, data aggregation, data access, and rendering/visualization, highlighting how these aspects are strictly connected. Section 3 describes all proper requirements for fast IoT data ingestion; here we have proposed the required evidence on differences within models and phases of the IoT device data ingestion process to facilitate the data rendering and analysis (thus, addressing the differences among IoT devices, KPIs, and POIs). Section 4 describes the data ingestion architecture and workflow, stressing the relationships with the Snap4City tools on top of which they have been developed. Section 5 presents the formal model for the IoT devices together with some reasoning processes which are needed to guarantee that the model is well-formed and the resulting data can be exploited by operators, providing rendering and views. In Section 6 it is shown how the formal model has been used to generate wizard-based mechanisms adopted for shortening the path for the visual production of a dashboard on Snap4City. The validation process has been based on validating all possible cases of mapping data to visual features via a dashboard wizard that proposes possible data results from operators and slices to the user with corresponding possible visual representations. Conclusions are drawn in Section 7.

## 2. Related Work

Several studies in the context of smart cities have shown architectures and frameworks addressing big data ingestion strategies (e.g., [13,17,18,19,20,21,22,23,24]). In [13] a smart city architecture, which includes a data aggregation layer to bring data to a knowledge base for the city, is defined for the execution of a wide range of data analytics as well as the formalization of smart city APIs to be accessed by the web, mobile apps, and smart city dashboards. 

The study in [17] presents methodologies to decrease the effort of implementing new smart city solutions and to support the sharing of components by encouraging developers to design their applications in a modular way so as to detect reusable IoT services, thus resulting in the implementation of 35 city services in 27 cities located in Europe and South Korea. The solution in [18] describes City Platform as a Service—Integrated and Open (CPaaS.io). It was a collaborative project between Japan and the EU to establish common smart city platforms for deployment in real smart city use scenarios to solve urban problems and support the applications cities deal with. In [19] an IoT-oriented data storage framework is introduced, integrating both structured (e.g., numbers and dates) and unstructured (e.g., video and audio) data. The research in [20] offers a smart city testbed for applications in different areas (e.g., intelligent transportation and pedestrian safety) to act as an open platform for testing sensors and algorithms. Furthermore, meta-models have been proposed to manage the heterogeneity of datasets to be processed in the context of big data (e.g., [25,26]). In [25], for example, an approach is proposed to deal with data pre-processing issues (e.g., collect, integrate, clean, merge, aggregate, and transform) of different data files from different resources by transforming their associated entity relationship (ER) data models into a uniform data model. However, the proposed approach is not flexible enough because the ER data models of IoT devices and files are typically not available. The authors in [26] proposed universal meta-modeling for the big data storage layer to address the problem of the diversity of existing solutions by presenting a shift from a generic big data storage layer meta-model, based on Hadoop [27], to a Cloudera [28] distribution storage layer. This was in contrast to a considerable amount of smart city solutions supporting only a single domain (e.g., air pollution monitoring [29], roadside assistance [30], smart parking [31], urban [32] and public [33] transportation, smart power grids [24], and smart meters [34,35]). Therefore, when it comes to a smart city solution it must be possible to ingest, store, and process any heterogeneous multi-dimensional data from different domains, with the possibility of defining new ones. In [21], an architecture for ingesting and analyzing IoT data that uses both real-time and historical data (e.g., weekly and monthly) is presented to provide context for real-time analysis, open source components (e.g., Node-RED, Apache Kafka, Apache Spark and OpenStack Swift) optimized for large-scale applications.

The main identified axes of analysis of the state of the art are related to single data models, data aggregation, data access, and data rendering/visualization. Even if they might appear as aspects to be addressed separately, in reality they are strongly interconnected.

As to data models, most of the IoT devices and city entities have defined their own data model where the different addressed variables and measures are named and typed. Therefore, a large plethora of models and formats has been produced. At present most of such models can be formalized in JSON (JavaScript Object Notation) and XML (Extensible Markup Language). On the other hand, despite the common technical format, data models can be strongly different in terms of variable names, data type, and thus the meaning of variables. In this regard a valuable effort is performed by FIWARE [36] (e.g., [13,17,18]) to define a data model for context information based on an information model using the notion of entities (e.g., sensors/actuators IoT, POIs) [37], each is provided with an ID, a type (e.g., AirQualityObserved), and context attributes including attribute name (e.g., coordinates), attribute type (e.g., location), and attribute value. In particular, FIWARE (Future Internet ware) has also chosen the JSON format in NGSI [38] (Next Generation Service Interfaces) to define models.

In the context of data aggregation there is the need of establishing relationships among data, taking into account what has already been ingested into the platform in terms of entities and data types. In smart city systems thousands of different data models may be integrated, as proposed by different vendors in different phases of smart city life. For example, an IoT device of a certain vendor may have slightly different measured variables with respect to the ones of a different vendor due to the evolution of technology and product differentiation. Global standardization could be a benefit, but would be hard to obtain in rapidly evolving conditions where legacy solutions and new components must be integrated. Data warehouse approaches can regularize data at ingestion time to make possible their quick exploitation (e.g., by establishing relationships and regularizing data so as to know data types) [11]. On the contrary, a data lake approach typically performs data ingestion without any processing and regularization; those aspects are left to data usage. When there are thousands of different models, data regularization at usage time is not efficient and becomes too complex to produce results in time. In fact, in the IoT field performing a certain data understanding and regularization during the ingestion phase is very difficult to be modeled and implemented when there are many data models. The regularization can be used in data warehouse and hybrid approaches, which perform a partial regularization to use specific data marts at the disposal of the rendering and visualization [39]. 

**Figure 1:**
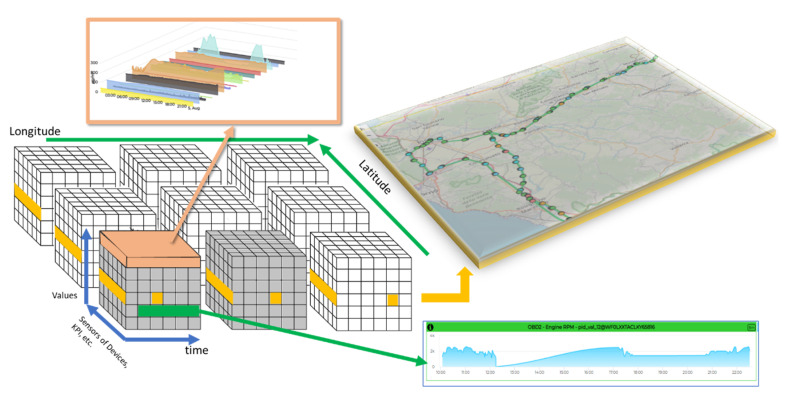
Example of hypercubes of IoT device data from the territory with their time series and variables, and some of the possible views: multi timeseries in 3D (pink), map multiple values on the same trajectories over time and territory (orange), and time trend of a time series (green).

As to the state-of-the-art analysis, its final aspect is data access and data rendering/visualization. These aspects must be addressed at the same time. OLAP (online analytical processing) [40] can be the starting point since each IoT device, associated with a data model and source, can be a hypercube with several axes. See for example Figure 1, which illustrates a hypercube with axes such as space (latitude and longitude), time, and variables with values of different kinds/units (e.g., numbers, enumerate, and strings). Figure 1 shows possible views: multi-series, multiple values on the same trajectories over time and territory, and time trend of a time series, which can be obtained from sliding or dicing the hypercube. These effects can be easily obtained if data are aligned in terms of characterization, geoinformation, and temporal values.

In addition, semantic relationships may exist among different hypercubes and over time. A suitable example would be modeling an IoT device collecting 10 measures and moving along the city (e.g., car or bus). Most IoT applications need to exploit a very large number of hypercubes, which can be regarded as data models at the same time in visualization and data analytics. For instance, consider the visualization of a dense heatmap of all temperatures in a region which can be produced based on thousands of device data, provided by different operators and different producers (thus involving many different kinds of processes, e.g., visualization, analytics, data-driven processes, security, and performance in complex queries as well as business intelligence queries). If data ingestion can rationalize all data into a common hypercube of several cubes, visualization tools can easily provide flexible views by sectioning the hypercube, including drill-down, drill-up, dicing, roll-up, and slicing [41], with a number of prepared queries.

Despite the above considerations, in most smart cities and IoT solutions data access is focused on accessing a single data element and time series without addressing the possibility of sectioning the hypercubes. Such limitations could be due to (i) the initial data ingestion model, (ii) difficulties in regularizing models in the aggregation processes, and (iii) the APIs providing the data access. In [22], the CitySDK Tourism API describes a solution to support access to information about POIs, events, and itineraries in the context of the CitySDK project [31].

While the discussed related works address several issues in smart city architectures, not one of the mentioned related works supports a data ingestion process using a user-friendly web-based environment described by a formal model, including a data model and a set of constraints (e.g., see the data structure uniformity in multi-sheet Excel files). Besides, they do not consider the management (i.e., feature creation, editing, and deletion) of IoT devices, including fixed and mobile ones, as well as POIs, using the loaded data. A new generation of smart city solutions is built on the fact that data can be provided by city users (e.g., citizens, commuters, and tourists). They are, at the same time, data providers and data consumers [42,43]. The discussed related works, however, do not consider (except [22], which supports CSV files (comma-separated values)) the possibility of ingesting data/models when they are provided in a static format (e.g., Excel). Moreover, such studies do not facilitate data validity assessment (e.g., the structure uniformity of all rows in the Excel file to be loaded, as well as the uniformity of each sheet compared to the other(s), missing mandatory data, and data type verification). The above-discussed related works (except [19]) do not focus on multi-tenancy support when the same data ingestion tool is used by multiple organizations with different users, where all such different users must be able to manage solely their own data. Finally, they do not provide an assistive process where the user is suitably directed during that data ingestion procedure (e.g., by providing guidelines and warning messages). The problems identified in the context of data warehouses are related to (i) the complexity of data structures to be ingested and verified during the ingestion process, (ii) the variety of data types and units to be collected (verified and exploited), (iii) the possible adaptation of the data kind dictionary, (iv) the costs and time needed for complex data ingestion, (v) the relationships with the initial data ingestion (data model registration), and (vi) the setup of data streams to continuously receive the registered data and models, just to name a few.

## 3. IoT Data Model Analysis and Requirements

In the context of smart cities and the IoT, there are many kinds of data to be collected and managed (e.g., POIs, IoT devices, heatmaps, flows, trajectories, matrices, and KPIs). On the other hand, the most storage-consuming data kinds are IoT devices with their time series, KPIs (which may also be represented as time series), and POIs (which are typically static information). Thus, IoT devices and KPIs to be added into a system have (i) metadata (structural and semantic descriptions and relationships), (ii) historical data, and (iii) real-time data streams. Furthermore, in most cases processes and models consuming that information are different, and this implies that all the above-mentioned aspects have to be harmonized with the rest of the data in the system in order to achieve efficient data exploitation and rendering. For this reason, this section performs an analysis of and summarizes the most relevant requirements that an IoT data ingestion platform for smart cities and the IoT/Industry 4.0 must satisfy to accelerate/automate data-gathering procedures, including model registration, data aggregation/harmonization, and data ingestion for both static data and time series. The following analysis is focused on modeling and ingestion, while it is assigning the management of communication protocols of the IoT world to the real-time ingestion that is performed with some specific tools as described hereafter [2,17,44].

In this regard, most of the solutions for data warehousing are based on producing ETL (extract, transform, load) processes. In these processes, data are ingested (in most cases in Pull), registered in terms of structure and model, transcoded to fit with the general model, and then saved into the storage [45]. In data lake solutions they are typically ingested/extracted and saved to be processed later according to their usage. The extract phase is typically working in Pull, which means that they are used to take the content with protocols such as REST Calls, WebServices, FTP (File Transfer Protocol), etc. An alternative solution to the usage of ETL/ELT visual programming tools (such as Pentaho Kettle, Karma, and NIFI (NiagaraFiles tool)) can be traditional programming by case (e.g., using Java or Python), which is time-consuming and also error-prone with respect to ETL approaches depending on how many developers and programmers contribute to the code implementation process due to their different experience levels, which are in continuous evolution.

The arrival of an IoT approach has radically changed the rules because data, which are provided/ingested by IoT brokers in Push, (real-time, event-driven) dynamically accept new data models since IoT brokers directly resend messages, using almost the same format as they received it, to subscribers (e.g., the storage, a visualizer, or other devices). In fact, IoT brokers in principle do not need to know more about the message data model. This approach can be easily integrated to feed data lake solutions that leave data aggregation to usage. On the other hand, an aggregation process is still needed for complex cyber–physical systems where data ingestion would take hundreds or thousands of different models and processes. Moreover, data ingestion problems remain unsolved when it comes to collecting historical data not provided by IoT brokers and, in most cases, such data have to be recovered by some gateway server that has collected them; this approach is a suitable task for ETL processes.

With the introduction of IoT technologies and concepts data warehouse solutions have been forced to radically change; somehow they have been obliged to grasp the whole ingestion process as an IoT data-gathering process. The main change has been the introduction of the IoT device concept, which may formalize the modeling of structured messages both from/to physical devices and virtual devices. Virtual devices can produce/receive messages from a user interface (i.e., dashboards, applications, synoptics, and processes [46]), as well as from databases, files, third-party services, etc. In the following, they are all modeled and called IoT devices.

Therefore, in the context of smart cities and Industry 4.0, the main data structures can be classified as:

- POI elements representing entities in maps (e.g., vista points, shops, banks, museums, and schools). They may present a relevant number of descriptive metadata of any kind, but not one of them is a time series and represents stable geolocations (static GPS coordinates).
- KPIs providing a value changing over time (a time series) and presenting a limited data and metadata structure. In some cases, they may be precisely geolocated (e.g., the daily revenue of a restaurant), in others they may be generically geolocalized in some representative point or attached to a city entity (e.g., the number of restaurants open in 2020 in a certain district or the weekly number of newborns in the city). The associated geolocation can be the center of the area (e.g., city, region, or city sector). In some cases, KPIs may provide values over time with variable GPS locations which make them similar to an IoT device apart from the lack of multiple variables, as described later.
- IoT devices which are typically geolocated, and their position can be stable or mobile (dynamic GPS values). They may present multiple static attributes and multiple variables changing their value over time, even non synchronously, with one another. Such variables create a substantial time series. IoT devices can be co-located or may refer to some POIs which can refer back to them.

All these elements may refer to several different city components, for example a road segment, a crossroad, a pillar, a building where they are installed, a civic number which they refer to, or a physical device (e.g., a pump or a pipe), thus contributing to the modeling of the so-called digital twin of a physical object which can be visually represented in a different manner on dashboards, mobile apps, etc.

The main differences among above-mentioned data elements are reported in Table 1. As can be observed IoT mobile devices provide geolocated changes over time and can be regarded as the most generic meta model where geolocation and variables may change over time. For example, a car could be represented by an IoT mobile device since it may move and provide some variables that change over time as a time series (e.g., engine regime, velocity, the status of the gasoline tank, position, and temperature). This also means that in the group/category of IoT mobile device (e.g., all the city busses) there is some information which is (i) identical for all city busses, (ii) specific for each bus, and (iii) detailed info changing over time and space. For example, the status of the tank, time measures related to internal temperature, and the number of people on board. This fact implies a complexity to be managed to prevent any useless saving of the same static values (as (ii) and (i)) multiple times in the storage at any message.

Thus, the category of IoT mobile device can be regarded as an abstraction of IoT devices and KPIs, while POIs should be treated in a different manner because they do not provide any time series. It could be regarded as a specialized version of an IoT mobile device, while the standardization of POI static attributes could shorten the activities of managing them on mobile applications and services for final users.

According to the above description, a distinction must be drawn between the IoT device model and its instances. In the context of the IoT, an IoT device model is a generic description/template, also called a data model or a smart data model, such as in FIWARE [36]. The actual IoT devices may be stamped out from an IoT device model. For example, the model of an IoT device of a smart bike rack presents a set of descriptive features: a type, a producer, an ID, and a set of variables with their definition. An actual IoT device of a bike rack may be, for instance, named BikeRack34, located in Madison Square. Thus, the IoT device model is used as a template to register an actual IoT device bike rack in a city location (e.g., with static attributes of the IoT device: ID=BK34, located at 11.25245:43.24324; variable values which may assume different values of time such as datetime = 2021/03/15T09:03, freeslot=12, and numbike = 3). The registration of new IoT devices is an important step in the IoT broker to make it ready to receive messages from/to a device and accept subscriptions to receive messages from other IoT network elements/consumers. IoT device registration could even be performed without using the IoT device model in most of the solutions, while the recent trend is to have data models as standard representations—for example on FIWARE and related solutions such as Snap4City [15]. Once an IoT device is registered it can send real-time data messages to the broker (e.g., a new message every 10 min). This implies that the IoT device must both be compliant with the broker in terms of protocol and have the correct authentication and information to communicate. Real-time data messages are single elements of time series of IoT device variables which may have GPS values moving/changing over time, thus resulting in the evidence of an IoT mobile device.

IoT devices also yield static attributes (see Table 1) which could be sent into each message (even if the consumption of bandwidth would not be recommended, while internet protocols in JSON and XML could permit that approach). Moreover, storing the same values for static attributes with each new message can be largely inefficient. Both storage and/or ingestion processes can filter static attributes belonging to both categories of device as well as single elements once their value is repeated. This work may also be performed by some IoT brokers (e.g., Orion Context Broker FIWARE) [36]. Therefore, the fact that POIs do not present time-varying variables implies that they do not produce messages, thus making it almost useless to ingest them via an IoT broker as IoT devices. The utility of having them on a broker could make sense only if the broker is used to report the context of the area, and thus it is set up to respond to some geoqueries.

Another relevant aspect is the characterization of dynamic variables in terms of their semantic meaning. In most cases a taxonomic classification could also be used. According to our terminology each variable should be characterized by at least three concepts for its correct usage: value type, value unit, and data type. The value type describes the semantics of data and facilitates the identification of the working scope of variables. For example, a variable with “Power” as a value type may have to accept as value units a limited number of different units of measurement, such as mW, KW, MW, etc. In some IoT solutions and protocols these aspects are not fully formalized (leaving them to the application level), thus making more difficult the integration of data coming from different device models and, in some cases, even from different devices instantiated from the same model, because the model does not provide a full characterization of variables. A suitable example here is considering a comparative graph of a time series of data when their units of measures are not known and may change at every message or can be diverse for different series. For instance, when measuring pollutants complexity is high because they may present as value units ppm (parts per million), ppb (parts per billion), μgm^3^ (micrograms for cube meter), as well as mgm^3^ (milligram per cube meter), which can be converted only by knowing the molecules’ weights. Moreover, variables need to be characterized also in terms of their coding and representation. At the level of data processing and storage it may make sense to know their data type representation as a technical coding. Following the example of pollutant density, it would make sense to have them coded by using as data type: float, and not: string, Boolean, GPS coordination, timestamp, etc.

### Requirements

According to our analysis of real-world scenarios many users who work with smart city platforms are non-technical people. For this reason, providing simple and assisted solutions for data ingestion is highly needed, which may start from tables in Excel files. As described above, an IoT data ingestion process implies addressing (i) IoT device modeling, (ii) IoT device instantiation/registration as well as the loading of historical data messages, and (iii) connection with real-time streams of data messages (with physical or virtual devices) arriving in the platform in Push from brokers, processes, dashboards, etc., or gathered in Pull from gateways. A tool to automate and simplify IoT data ingestion phases would also be a simplification of the above-described ingestion phases. Therefore, to reduce the ingestion time and increase the quality of the data ingestion processes we have addressed the problem by means of an automated solution for the so-called early data ingestion, making it usable for both technical and non-technical personnel. This approach is called early data ingestion since it can also be used by skilled personnel for shortening the phases to prepare the IoT platform to receive real-time data from other processes. In fact, once IoT devices are registered both IoT broker and platform are ready to receive historical and real-time data. The latter can be directly provided by IoT devices to the subscribed services, while historical data must be recovered from some server/gateway in Pull or loaded with some files or database. These last two processes are time-consuming because their setup implies creating ad hoc programs, data flows, and sometimes ETL processes.

The following list includes the main functional and non-functional requirements the ideal ingestion solution should have in order to automate the above-described phases. Therefore, the so-called data loader solution for early data ingestion must provide assistance to users semiautomatically in the data ingestion process when data is loaded by using Excel files. The system will have to:

- Understand data loaded to identify and create an IoT device model, which can be performed programmatically or from a graphical user interface by providing several pieces of detailed technical information, which is not an easy task for non-technical personnel;
- Understand data loaded to identify and register IoT devices (which are instances of the IoT device model in specific contexts: positions, street name, city location, etc.);
- Understand data loaded to identify time series of IoT devices, storing time series values of historical data related to IoT devices;
- Set up the needed authorizations to obtain access to the data table and create an IoT device model, IoT devices, and data into the Snap4City infrastructure [15];
- Ingest data modeled as hypercubes including a set of variables of different kinds and corresponding values which may typically change over space (as latitude and longitude) and time;
- Connect data ingested with other data entities already available in the storage and with the general model according to a dictionary of value types, value units, and data types. In fact, without proper characterization the data ingestion can create incorrect visualization in dashboards and/or on data analytics due to possible wrong data operations (e.g., mean, addition, difference, training and median);
- Manage incoming data based on the same tenant/organization of the user performing the loading, always respecting the user’s privacy and GDPR;
- Comply with a model to support the visualization of data coming from different hypercubes on the same representations if they have compatible characterizations in terms of geospace and time.

Even if data rendering is performed using Snap4City dashboard builder [15], non-regularized data ingestion would create difficulties in the rendering and force the dashboard designer to create a transformation in the business logic of the front end. This is what happens in most cases with business intelligence tools on top of data lakes, which leads to much time and space being spent to match data into each visualization model by repeating the same activities several times.

In addition, such a solution must satisfy non-functional requirements. This data ingestion procedure must be convenient enough to use by bypassing low-level details and steps (e.g., manual data modeling registration, data kind verification, creation of data instances) when addressing POIs or IoT devices and their time series.

The ingested data have to be managed to respect both privacy and GDPR rules on privacy [16]. In this case, to be GDPR-compliant means at least to (i) keep user data separate, for example by creating distinct tables and databases in addition to protecting them from unauthorized access, (ii) encrypt personal data, (iii) allow users to delete their uploaded data at any time, (iv) upload data as private by default, and (v) log and audit any access to the data, etc. 

## 4. General Architecture and Workflow

**Figure 2:**
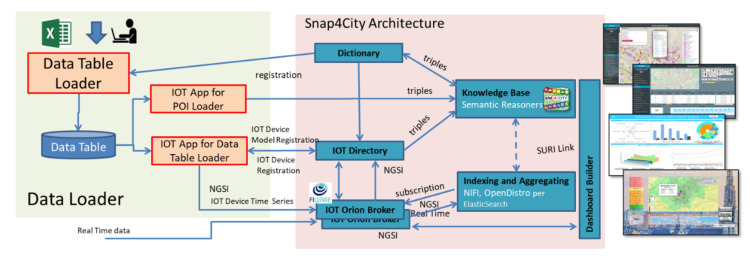
Data loader architecture with respect to Snap4City main components.

According to the above-described requirements for a solution for early data ingestion we have defined and implemented a data loader on top of the Snap4City infrastructure [15] and open-source solution. Figure 2 shows the architecture where Snap4City [15] tools are distinguished with respect to the implementation of the data loader described in this paper. Before designing and developing the data loader, data ingestion on Snap4City [15] was based on developing ad hoc programs in Node-RED, ETL Pentaho Kettle, or by using specific programs in Java or Python and exploiting the API to create IoT devices and load their instances. The IoT directory exploits the dictionary during the creation of an IoT device model or IoT devices. Moreover, the dictionary is exploited by the knowledge base as a vocabulary of possible values and their relationships among value types, value units, and data types.

**Figure 3:**
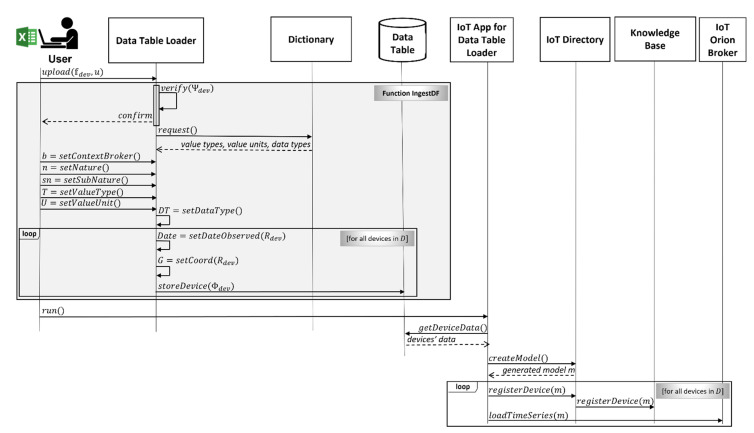
Data production process, from Excel file to IoT devices and their instances.

The data loader is based on four main components supporting the above-listed requirements, and it is described in the following section. The whole process is described by the sequence diagram reported in Figure 3. It clarifies interactions among different entities: user, data table loader, data table, and IoT app, with respect to the former Snap4City architecture components.

The data table loader is the main assistive tool assisting in the input of a number of Excel file formats (with single and multiple sheets) and transforming raw data into a well-formed data table by exploiting a dedicated rule-based system. The data table loader’s task is to:

1. Recognize data ingestion structures and understand Excel format types;
2. Verify the correctness of the related ingestion format;
3. Identify the presence of time series (providing a timestamp per instance);
4. Identify the presence of GPS coordinates (which could be per instance, per device, or missing);
5. Identify value type, value unit, and data type according to the dictionary;
6. Create a well-formed data table for the automated process of deployment and data loading.

The data table loader analyzes/verifies the Excel file to identify the presence of IoT device models (for static and moving devices) to be used as a template for producing IoT devices and corresponding time series (which in turn are time instances of the IoT devices). Once the file analysis has been performed, the data table loader guides users to provide possible missing information. This approach allows for a correct ingestion of the data. Specifically, when the user is not a specialist in data ingestion it is absolutely important that s/he is supported by an assistant process. The first phase is therefore supervised and guided to map provided data into concepts and types of the Km4City multi-ontology. The tool in fact connects data with classes of Km4City ontology and characterizes data according to the provided dictionary. For these reasons the user is assisted in his/her “walk through” ingestion process by a wizard which suggests, verifies, and almost automatizes all the complex steps of a formal process (as described hereunder). The process also provides warnings and training suggestions, which can be useful for shortening successive loadings.

The data table loader supports a formal model to ingest and regularize different data hypercubes related to different data models. To this end, it adopts a set of rules to identify data patterns, transform non-well-formed structures, define/assign missing data, and make automated transformations, for example performing georeverse (from geoinformation as area and street, civic number to GPS) [47]. When missing data (e.g., GPS, date, and time) are identified the user is assisted in providing them (e.g., the broker ID, classification of data as nature and subnature, and variable characterizations for which a model is provided, if not explicit in the Excel file). The data must be carefully verified during the ingestion process. Such verification should address a range of aspects. In addition to typical verification schemes (e.g., verification of file type, characters in the file name, or content) there are pre-defined reasoning rules (constraints) that must be followed to verify the compatibility of loading data with the designed and implemented platform. For example, considering a multiple-sheet Excel file, the structure (e.g., number of columns, column header names, column data types) of data in all sheets must be the same. The verification rules on reasoning are discussed in detail in Section 5.

Once the data table is generated, specific IoT apps are activated to commit the actions on the Snap4City platform [15]. IoT apps address separate processes for ingesting IoT devices and POIs. These processes, which are implemented as Node-RED (actually Node-RED plus Snap4City MicroService Libraries), get access to the users’ data table and start an automated process. It includes (according to the identified data): (i) the creation of one or more IoT device models, (ii) the registering of IoT device instances, and (iii) loading data messages of time series of the IoT device’s corresponding historical data.

The data loader allows any data loading by different users belonging to different organizations. Each organization, which is practically a tenant in the solution, determines a set of geoareas, a different group of users, a private data space, etc. The data loading respects the user’s privacy and GDPR according to what has been described in Section “*Requirements*”. Supporting multitenancy is required in order to grant that each organization processes, views, or edits only its own data, typically by customizing an IoT app for data loading (for IoT device data and POIs). In addition, the process must produce models, entities, and time series data belonging to the user who uploaded them and to the user’s organization. Therefore, data loaded into data table are private, they belong to the user who loads them. Only specific authorized agents/users can access the loaded data by the data loader, ingest them via an IoT app to register devices/POIs, and load them into the storage. Thus, each organization has a different IoT app for data loading owned by one of the authorized users for content ingestion.

**Figure 4:**
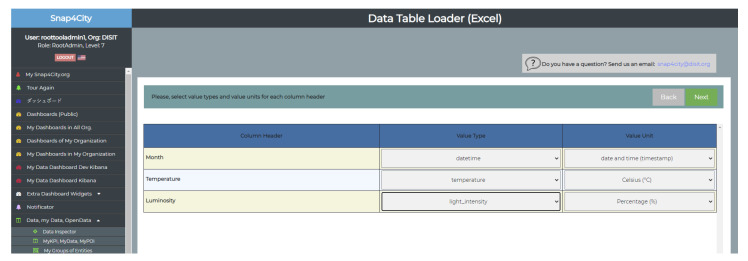
User interface of the data table loader in the phase of characterization of variables.

Figure 4 shows the user interface of the data table loader. The screenshot is taken in the phase when value types and the value units are associated with IoT device model features (see the column headed *Column Header*), while data types are provided automatically on the basis of the dictionary. Access to the data table loader, implemented in Python, is granted to authorized users. In addition, for each authorized user IoT apps are also provided as Docker containers, executed on the cloud-based scalable Snap4City platform [15]. The cloud-based Snap4City IoT apps are managed on a cluster of Marathon Mesos virtual machines (VMs), each of which may manage up to 70 IoT Apps. When more IoT apps are needed a new VM is deployed [44].

**Figure 5:**
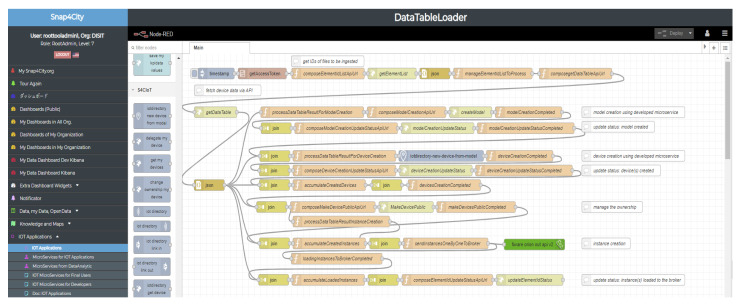
IoT apps for the data table loader. The figure is a snapshot of the tool user interface on the Snap4City environment which includes web programming in Node-RED plus the Snap4City library of microservice nodes.

The IoT app for the data table loader analyzes the data table to (see Figure 2 and Figure 3) (i) create one or more IoT device models into the IoT directory (i.e., the administrative tool for managing all IoT brokers, device models, and devices), (ii) register, via the API, IoT devices templates from the formerly generated model into the IoT directory (which, in turn, registers them into the knowledge base), and finally (iii) prepare and send messages to IoT brokers (FIWARE in NGSI) to load historical time series data which were in the Excel file. Once an IoT device is created, real-time data may arrive, and they are automatically ingested into the storage based on Open Distro for Elasticsearch. Figure 5 reports a snapshot of an IoT app for the data table loader which has been developed in Node-RED using Snap4City libraries [48]. The IoT app can be easily read as follows: It periodically gets access to the data via a getDataTable API call. The resulting information is transformed in a JSON format. Then, on the basis of collected data, the IoT app performs the following sequence of operations: (i) creation of an IoT device model, updating the data table status; (ii) creates IoT devices and updates the data table status with this new step; (iii) manages the ownership of the device; (iv) creates all the time series instances for each created IoT device; and finally (v) updates the information into the IoT directory of Snap4City to make IoT devices managed by the system. The join blocks are used to maintain the information accessible in the data flow and avoid temporary variables.

The IoT app for the POIs loader takes data representing POIs from the data table and generates the needed semantic triples to be loaded into the Snap4City knowledge base, KB, which is a Virtuoso RDF store. Triples connect the new POIs with the other entities of the KB to allow semantic queries to be performed as those performed by smart city APIs and dashboard builder tools are. IoT apps are quite simple, since a certain number of microservices has been developed to automate some of the more complex processes, such as (i) creating an IoT device from IoT device models, (ii) sending NGSI V2 messages to the IoT Orion broker, (iii) recovering information about an IoT device, (iv) recovering a list of IoT devices belonging to the user, (v) posing queries to the knowledge base via smart city APIs to discover/recover relationships, etc.

IoT devices/POIs are registered into the IoT directory, the Orion broker, and the knowledge base. Thus, they immediately become usable for end users as data sources for (i) Orion broker NGSI API usage, (ii) smart city APIs, (iii) the dashboard builder, which also exploits the integrated data model on the basis of value type, value unit, and data type, as described in the following, (iv) data analytics/reasoning as well as for other data transformations/reuse if needed, and (v) any other possible IoT app processes as to data transformation or business logic (e.g., business intelligence, computing average, descriptive statistics, forecast, and anomaly detection [48]). We have addressed data reasoning since the models registered into the knowledge base can be used for spatial, relational, and temporal reasoning, while time series are stored into the Open Distro for Elasticsearch.

The following sections describe how IoT mobile devices (being the most generic data structure in the IoT domain) and POIs are modeled and formalized in the proposed solution. In our approach both IoT and POI models generally include two categories: (i) data models, which represent features, their relations, and other attributes (e.g., context broker or category) in a given domain and (ii) rules of reasonings, which describe a set of constraints to be imposed to ensure that data is suitably and correctly ingested, considering the proposed solution. 

## 5. Formal Model for IoT Mobile Devices

We have taken into account a scenario where an Excel file, ${𝕗}_{dev}$, with a set, S, of sheets contains data associated with a set, D, of IoT devices to be modeled, registered, and ingested (in most cases). As a general rule, in the file, ${𝕗}_{dev}$, captured values associated with each device in D must be placed in a separate sheet, s∈S. Additionally, column headers in each sheet, s, are placed in the first row and defined in the vector, $H_{s}$, of column headers. All sheets in *S* must have the same column headers with the same order, i.e., $H_{s_{i}}=H_{s_{j}}=H,s_{i},s_{j}\in S$. On the contrary, if column headers in two or more sheets describe different kinds of data, the file, ${𝕗}_{dev}$, can be split into two or more files. Each row in the file, ${𝕗}_{dev}$, represents an instance of a device in D. In the proposed solution, a device data model, $\Phi_{dev}$, associated with the devices in D is identified by the tuple $\Phi_{dev}=\langle ID,SURI,\;F,T,U,DT,V,G,Date,\;n,sn,u,o,b\rangle$ where:

- *ID* is used for IoT device identification;
- *SURI* is used to univocally identify the IoT device according to linked data;
- F is the set of features of IoT devices in D which includes members of column header vector *H*. For example, F={Arrival city, Departing country,Arrivals,Overnights};
- $T=\{\left(f,t_{f}\right)\;|\;f\in F\}$ is the set of feature–value type pairs where the pair, $\left(f,t_{f}\right)$, associates the value type, $t_{f}$, with the feature, f. For example, the feature *Arrivals* could be associated with the value type *people count*;
- $U=\{\left(f,u_{f}\right)\;|\;f\in F\}$ is the set of feature–value unit pairs where the pair, $\left(f,u_{f}\right)$, associates the value unit, $u_{f}$, with the feature, f. For example, the feature *Arrivals* could be associated with the value unit *number*;
- $DT=\{\left(f,dt_{f}\right)\;|\;f\in F\}$ is the set of feature–data type pairs where the pair, $\left(f,dt_{f}\right)$, associates the data type, $u_{f}$, with the feature, f. For example, the feature *Arrivals* is associated with the data type *integer*;
- $V_{+}=\{\left(f,v_{f}\right)\;|\;f\in F\}\subseteq V$ is the set of feature–value pairs where the pair, $\left(f,v_{f}\right)$, associates the value, $v_{f}$, with the feature, f, which represents an instance of the device, d∈D. Back to our running example, $V_{+}=\left\{\left(Arrival\;city,Mostar\right),\left(Departing\;country,Italy\right),\left(Arrivals,2135\right),\left(Overnights,2775\right)\right\}$ is an instance, $V_{+}$, which includes the captured values of the device, d∈D;
- G$=\{\left(V_{+},g\right)\;|\;V_{+}\subseteq V\}\subset W=\left[-180,180\right]\times \left[-90,90\right]$ is a subset of WGS84 (World Geodetic System 1984) geographic coordinates where g describes the location of capturing the instance, $V_{+}$, by the IoT device, d∈D;
- $Date=\{\left(V_{+},date\right)\;|\;V_{+}\subseteq V\}$ set includes where date denotes when the IoT device, d∈D, captured the instance, $V_{+}$;
- n denotes the nature/category (e.g., tourism service) of IoT devices in D;
- sn denotes the subnature/category (e.g., travel information, tourist trail) of IoT devices in D associated with the nature, n;
- u is the name of the user uploading the file, ${𝕗}_{dev}$;
- o denotes the organization of the user, u;
- b denotes the associated context broker of IoT devices in D.

Additionally, datasets are verified during the ingestion process by applying a set, $\Psi_{dev}$, of verification rules to ensure that data to be ingested are compliant with the designed ingestion solution. For example, if there is a date of observation, date, for each instance, $V_{+}\subseteq V$, of the device, d∈D, then date must follow the ISO 8601 standard, i.e., YYYY−MM−DDThh:mmTZD (e.g., 2020−07−16T19:20+01:00). Another example of a device verification rule in the set, $\Psi_{dev}$, is that special characters (e.g., blank space, #) are not allowed in the name of the file, ${𝕗}_{dev}$, the sheet names in S, and the column headers in H. Additionally, a set, $R_{dev}$, of rules of reasoning describes the logic of the data ingestion process for IoT devices, and as a result it allows data to be ingested in a uniform, logical way. Considering each device, d∈D, the most relevant rules of reasoning in the set, $\Psi_{dev}\;$, are defined as follows:

- If, for each instance, $V_{+}\subseteq V$, of d∈D there is not a date of observation, date, then a single date of observation, 𝕕𝕒𝕥𝕖, is assigned by the user to all device instances in D, i.e., if $\forall \;V_{+}\subseteq V,\;∄\left(V_{+},date\right)\in Date\to !\exists 𝕕,\;\left(V_{+},𝕕𝕒𝕥𝕖\right)\in Date$;
- If, for each instance, $V_{+}\subseteq V$, of d∈D (i) there is not a coordinate, g, and (ii) there is an address, a, then a possible coordinate, $\overset{´}{g}$, is assigned to $V_{+}\subseteq V$ by exploiting our ServiceMap service [49], i.e., if $\forall \;V_{+}\subseteq V,\;∄\left(V_{+},g\right)\in G\wedge \exists a\to \exists \overset{´}{g},\;\left(V_{+},\overset{´}{g}\right)\in G$;
- If, for each instance, $V_{+}\subseteq V$, of d∈D (i) there is not a coordinate, g, (ii) there is an address, a, and (iii) no coordinate, $\overset{´}{g}$, is found by exploiting our ServiceMap service [49], then the instance, $V_{+}$, and the pair, $\left(V_{+},date\right)$, are, respectively, removed from V and Date, i.e., if $\forall V_{+}\subseteq V,\;∄\left(V_{+},g\right)\in G\;\wedge \exists a\wedge ∄\left(V_{+},\overset{´}{g}\right)\in G\to V=V\backslash V_{+}\wedge Date=Date\backslash \left(V_{+},date\right)$;
- If, for each instance, $V_{+}\subseteq V$, of d∈D (i) there is not a coordinate, g, and (ii) there is not an address, a, then a single coordinate, 𝕘, is assigned by the user to all device instances, i.e., if $\forall \;V_{+}\subseteq V\;∄\left(V_{+},g\right)\in G\wedge \;∄a\to !\exists 𝕘\;,\left(V_{+},𝕘\right)\in G$.

The procedure of device data ingestion in the proposed solution (see Figure 3) can be shortly described by a pseudocode, as presented in Algorithm 1. First, the Excel file, ${𝕗}_{dev}$, uploaded by the user, u, is verified, considering the set, $\Psi_{dev}$, of verification rules. If some error(s)/warning(s) arise(s) the file loading procedure is blocked and informative guidelines are supplied to handle the error(s)/warning(s). Otherwise, while the data type set, DT, is automatically detected and set by the data table loader, the context broker, b, nature, n, subnature, sn, value type set, T, and value unit set, U, are set by the user. Then, considering the set, $R_{dev}\;$, of rules of reasoning, a date of observation and a coordinate is calculated and assigned to each instance, $V_{+}\subseteq V$, of each device, d∈D, and they are added to the sets Date and G, respectively.
**Algorithm 1.** The pseudocode briefly describing the proposed procedure of device data ingestion.1: Function ingestDF (${𝕗}_{dev}$)2: //verify the file ${𝕗}_{dev}$ according to verification rules defined in $\Psi_{dev}$3: verification_warning: =verify (${𝕗}_{dev}$);4: If (verification_warning==∅) do {5: // set by the user6: b:=setContextBroker();7: n:=setNature();8: sn:=setSubNature();9: T:=setValueType();10: U:=setValueUnit();11:  12: //automatically set by the Data Table Loader13:                DT:=setDataType();14:  15: For each (*𝑑*∈*𝐷*) do {16: // dateObserved setup17:                   Date≔∅;18: If ($\forall \;V_{+}\subseteq V\;,\;\exists date$) do {19: // if, for each instance $V_{+}\subseteq V,$ there is an observation date d20:                $Date:=Date\cup \left(V_{+},date\right);$21: } else {22: // a single date of observation 𝕕𝕒𝕥𝕖 is assigned, by the user, to each instance $V_{+}\subseteq V$23:                $Date:=Date\cup \left(V_{+},𝕕𝕒𝕥𝕖\right);$24: }25:  26: //coordination setup27:                    G≔∅;28: // for each instance $V_{+}\subseteq V$ if there is a coordinate g29: If ($\forall \;V_{+}\subseteq V,\;\exists g\;$) do {30: $G≔G\cup \left(V_{+},g\right)$;31: // for each instance $V_{+}\subseteq V$ if there is an address a32: } else If ($\exists a,\;\forall \;V_{+}\subseteq V$) do {33: // for each instance $V_{+}\subseteq V,$ coordinate $\overset{´}{g}$ is calculated by the serviceMap34:                 $\overset{´}{g}\;≔ServiceMap\left(a\right);$35: // if $\overset{´}{g}$ is null, the association of the instance $V_{+}$ with the device d is removed36: If ($\overset{´}{g}==\mathrm{null}$) do {37:                   $V≔V\backslash V_{+};\;$38: $Date≔Date\backslash \left(V_{+},date\right)$;39: } else {40: $G≔G\cup \left(V_{+},\overset{´}{g}\right)$;41: }42: // If, for each instance $V_{+}\subseteq V$, there is neither a coordinate g nor an address a43: } else {44: // a single coordinate assigned, by the user, to $V_{+}\subseteq V$45:                  $G:=G\;\cup \left(V_{+},𝕘\right);$46: }47: }48: }49: End function

### Formal Model for POIs

To model POIs we have considered a scenario where the file, ${𝕗}_{poi}$, contains data associated with a set, P, of POIs to be ingested. In real-world data ingestion scenarios, POI datasets can include a large number of features in different formats and data types, which could affect the possibility of providing a uniform POI data model to simplify their management on maps or in mobile apps. To mitigate the issue, we have proposed a template vector, ℱ, of features to be followed when POI datasets are generated. It contains 27 features (e.g., name, url, latitude, and longitude) that a POI may possess. In the file, ${𝕗}_{\mathrm{poi}}$, such features, as defined in the template vector, ℱ, are placed in the first row as column headers. Additionally, for each POI, p∈P, values associated with features in the template vector, ℱ, are considered in a separate row. A POI data model is then identified by the tuple, $\Phi_{poi}=ℐD,SUℛℐ,V,ℊ,𝓃,𝓈𝓃,𝓊,\;ℴ,𝒷$, where:

- ℐD is used for POI identification;
- SUℛℐ  is used to univocally identify the POI according to linked data;
- V is the vector of values where $V_{𝒻}$ denotes the value associated with the feature, 𝒻;
- ℊ ⊂W=[−180,180]×[−90,90] is a WGS84 (World Geodetic System 1984) geographic coordinate which describes the location of the POI, p∈P, described by *latitude* and *longitude* features and presented in the associated columns;
- 𝓃 denotes the nature/category (e.g., tourism service) of POIs in P;
- 𝓈𝓃 denotes the subnature/category (e.g., travel information, tourist trail) of POIs in P associated with the nature, n;
- 𝓊 is the name of the user uploading the file, ${𝕗}_{poi}$;
- ℴ denotes the organization associated with the user, 𝓊;
- 𝒷 denotes the associated context broker of POIs in P.

Besides, apart from a set, $\Psi_{poi}$, of verification rules (e.g., special characters are not allowed in the file name or cells of the file, ${𝕗}_{poi}$), a set, $R_{poi}$, of rules of reasoning is defined to describe the logic of POI data ingestion in our proposed solution. This is done to ensure the compatibility of POI data to be ingested with the designed solution. The most relevant rules of reasoning for POIs, defined in $R_{poi},$ are as follows:

- If, for each POI, p∈P, there is not a coordinate, ℊ, then an address must be provided to find possible coordinates, $\overset{´}{ℊ}$, for p∈P using our ServiceMap service [49]. To do so, values associated with features *province*, *city*, *streetAddress*, and *civicNumber*, which represent the address of the POI, p∈P, must be available, i.e., if $\forall p\in P,\;∄\Phi_{poi}^{\left(p\right)}\left(ℊ\right)\to \left|V_{province}\right|\neq \emptyset \wedge \left|V_{city}\right|\neq \emptyset \wedge \left|V_{streetAddress}\right|\neq \emptyset \wedge \left|V_{civicNumber}\right|\neq \emptyset$;
- If, for each POI in P (i) there is not a coordinate, ℊ, and (ii) there is not a coordinate, $\overset{´}{ℊ}$, found by the ServiceMap service for a POI, p, then p is removed from the POI-ingesting dataset, i.e., $\forall p\in P,\;∄\Phi_{poi}^{\left(p\right)}\left(ℊ\right)\wedge \;\exists p\in P,{\;\Phi }_{poi}^{\left(p\right)}\left(\overset{´}{ℊ}\right)=\emptyset \to P=P\backslash p$.

## 6. Validation: From Formal Model to Data Rendering

As above mentioned, a data ingestion process able to convert provided data into a common model can make the rendering process easier according to different views, slices of the model, etc. (for example, see Figure 1). In the paper, an automated ingestion process supported by a formal model has been presented to bring data into storage. As a result of the ingestion process formalized in previous sections, collected data can be uniformly queried and visualized in the Snap4City platform [15] by exploiting visual tools such as dashboards by choosing among a set of possible and suitable graphical widgets and charts according to the kind of data to be represented. For instance, a map widget can be suitable for showing POIs as well as the geographical position of IoT devices and IoT mobile device on the map as trajectories (see Figure 1). Moreover, time series charts attached to any variable can also be visualized by using widgets such as time trends, multi-series, calendars, and multi-data maps. On the other hand, widgets such as bars histograms, pie charts/donuts, and Kiviat/spidernet may be appropriate to represent time slice data as well as static data.

**Figure 6:**
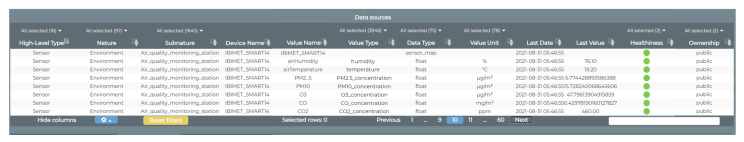
Overview of the Snap4City Dashboard Wizard interface.

Therefore, to assist the user in managing the complexity of the ingested data modeled as hypercubes we have created a tool which simplifies the first step in the process of mapping data to visual representations. This means that users can select data, and the tool (named Dashboard Wizard) provides a number of possible visual representations. This is not a limitation since users can also perform data transformations by using IoT apps if needed. The wizard assists users to select the desired data to be visualized and the most suitable graphical widget to represent them. In Figure 6 the Snap4City Dashboard Wizard search table is represented, showing the capabilities to search and filter by all the tuple components as described in Section 5 for the device data model, $\Phi_{dev}$, and the POI data model, $\Phi_{poi}$, in Section “*Formal Model for POIs*”. In other words, the whole set of data is preprocessed to extract possible views from hypercubes of cubes, and then they are made accessible on the wizard search table for faceted search.

In the wizard search table different data sources are represented by a specific high-level type (*HLT*) (e.g., POI, IoT device, mobile device, data table, and KPI). Then, nature, n, and subnature, sn, represent information about IoT, POI, or any other data source semantic category. Moreover, regarding IoT devices further information is shown, such as the feature name (f∈F, represented by the “value name” column in the Dashboard Wizard table, as shown in Figure 6), value type, $t_{f}$, value unit, $u_{f}$, data type, $dt_{f}$, the last value, $v_{f}^{last}$, and the last date–time, $date^{last}$. Information about both the user, u , and the organization, (o), are automatically handled depending on the logged user account, and this determines which data can be viewed in Dashboard Wizard loading (that is, public data as well as logged user’s own and delegated data).

The Dashboard Wizard (search table) is a useful representation to understand how the proposed data models help users in performing OLAP operations on data hypercubes, facilitating instance slicing; actually, as an example, each feature for an IoT device is ingested and thus represented separately. Therefore, many solutions are possible: from choosing and visualizing a single feature for a single device or any other data source (using so-called single-data widgets such as a gauge, speedometer, single content, etc.), to visualizing multiple features for several devices (using so-called multi-data widgets such as bar series, pie charts, Kiviat (spidernet), multi time-series, 3D data cubes, etc.). A general entry is also represented in the Dashboard Wizard for each device (that is, the first row which can be noted in the Dashboard Wizard table in Figure 6, having “sensor_map” as the data type). This is typically used to represent the general device, for example, on the multi-data map widget as well as on the OLAP 3D data cube widget, which is a 3D representation to be described in more detail in the following example.

### Validation Process

For the validation of the model, we have identified a large number of representations and produced a number of Excel files on which data providers are expected to perform a set of possible operations as described in Section 2 and Section 3. In Table 2, the most relevant cases have been reported. The columns represent both features and operations which have to be represented and performed on different ingested data, while the rows present different visual representations that have been validated to be produced, just selecting them as available tools listed in the wizard. For example, one of the features (the first column) represents the need of presenting data with the same value type (in this case it is set to “single”) even if they may come from different devices; on the other hand, the visualization of multiple data with multiple value types can be obtained (in this case it is set to “multi”). Please note that, for widgets such as bar series and donut, it is possible to apply an operator to exchange the value type and value units on the rendering axes. The solution has been validated in the context of the HERIT-DATA Interreg project with six different pilots in Europe (Florence in Italy, Pont du Gard in France, Mostar in Bosnia–Herzegovina, Dubrovnik in Croatia, Patra in Greece, and Valencia in Spain). They have provided a large number of datasets in Excel files regarding city evolution, people flows, and tourism. Therefore, many heterogeneous data coming from several operators have been ingested and aggregated to provide representative dashboards for city operators who can exploit them according to their business intelligence. A validation process has been performed by verifying the possibility of obtaining in a few steps all possible representations and demanded features from the ingested data without any data transformation. In the following, some examples of dashboards are shown to represent data gathered through the data ingestion tool, as presented in this paper, illustrating how the proposed formal models can facilitate the management and visualization of complex and multi-dimensional data sources. In the following, the same notation used for describing the device data model, $\Phi_{dev}$, and the POI data model, $\Phi_{poi}$, in Section 5 is adopted.

The validation process was completed in 2020 and subsequently this solution was made accessible to Snap4City users, which developed a large number of dashboards and solutions. Table 3 provides a numerical overview of different data sources ingested in the Snap4City platform [15], which are represented by Dashboard Wizard distinct instances. Data sources are aggregated by HLT as described above, and for the different device-related HTLs (ETL sensor, IoT, IoT mobile device, and data table virtual devices) further details are provided, specifying the total number of ingested devices and the total number of device attributes.

**Figure 7:**
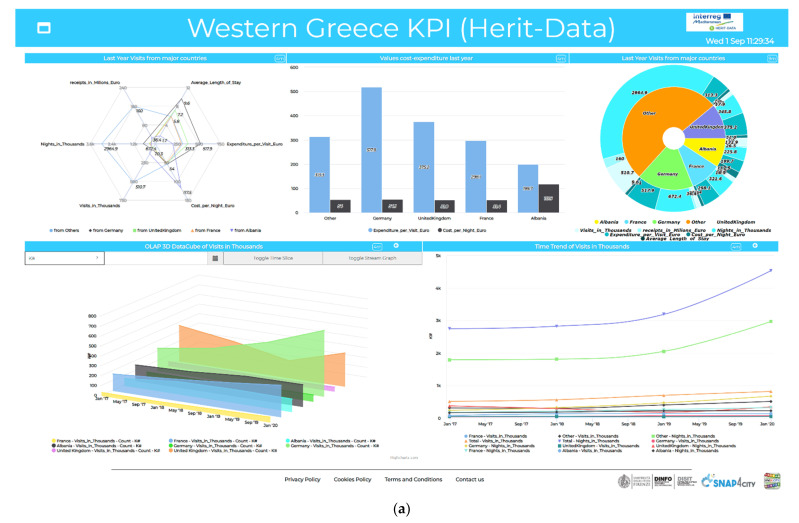

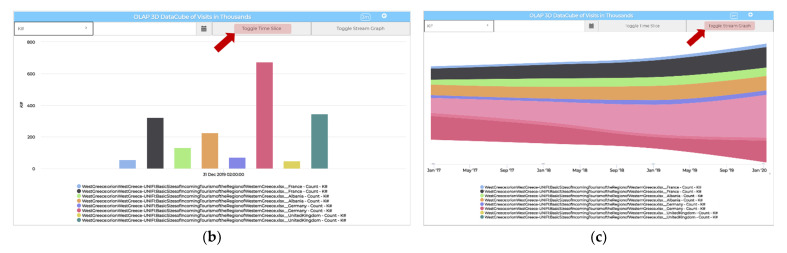
(**a**) Dashboards representing data collected by exploiting the data ingestion tool according to the Φ*_dev_* formal model: KPIs related to tourism flows data in western Greece; (**b**) details of OLAP data cube widget time slicing; and (**c**) stream graph.

In Figure 7a a dashboard visualizing some KPIs related to tourism flows data in western Greece is depicted. This dashboard exploits widgets such as histograms, pie charts, Kiviat, multi time series, and 3D data cubes to visualize KPIs describing the number of visits and nights, as well as cost expenditures for tourists coming to western Greece from foreign countries. These data were ingested and modeled as virtual IoT device data, i.e., from ${𝕗}_{dev}$ Excel data sheet files according to the formal model described in Section 5. Widgets used in this dashboard have been chosen to provide different representations. For instance, the Kiviat, bar series, and pie chart widgets are useful to represent aggregated views of the last values of different features for each device, thus showing the last value, $v_{f}^{last}$, for each feature, f, aggregated for each of the represented devices. The multi time series widget in the bottom right of the dashboard depicted in Figure 7a is useful to show the temporal trend of values collected for each feature of all the devices. Therefore this widget shows the set of values by dates for each feature of all the represented devices, i.e., $Date=\{\left(V_{+},date\right)\;|\;V_{+}\subseteq V\}$, where $V_{+}$ represents, in turn, the feature–value pairs (instances), i.e., $V_{+}=\{\left(f,v_{f}\right)\;|\;f\in F\}\subseteq V$, according to the $\Phi_{dev}$ model description provided in Section 5. Finally, in the bottom left of the dashboard in Figure 7a we have a 3D data cube widget, which is very useful to provide a multi-dimensional hypercube representation of data. This widget can take, as input, a general entry for an IoT device (such as, for example, the first row shown in the Dashboard Wizard table, as depicted in Figure 6), and not necessarily the device-selected features (i.e., the specific “value names” from the Dashboard Wizard), since it is instead required when creating the afore-mentioned widgets. In fact, the 3D data cube widget can automatically represent the whole feature set, F, of a certain device, aggregated by the value unit, $u_{f}$. The value unit can be chosen from a drop-down selection in the upper-left corner of the widget, thus providing different visualizations for the features associated with each different value unit. In the present case, as illustrated in Figure 7b, the vertical axis represents the amplitude (# count) while the horizontal axes represent the date–time and the device ID. This is similar to the view of the multi time series widget; however, the 3D data cube also allows OLAP operations to be performed. For instance, it is possible to perform time slicing by selecting on the calendar date picker a specific date and time and then clicking on the “Toggle Time Slice” button (as shown in Figure 7b). Drill-down and roll-up on time can be performed in edit mode by selecting different time ranges for data visualization. Once a time range for visualization is selected, it is possible to navigate different temporal ranges of data by using the arrow buttons in the top-left corner of the widget (this feature is also provided by the multi time series widget). In addition, a stream graph view is allowed by clicking on the “Toggle Stream Graph” button (as shown in Figure 7c), giving an overview of the varying amplitude of the different features for the different devices by time.

**Figure 8:**
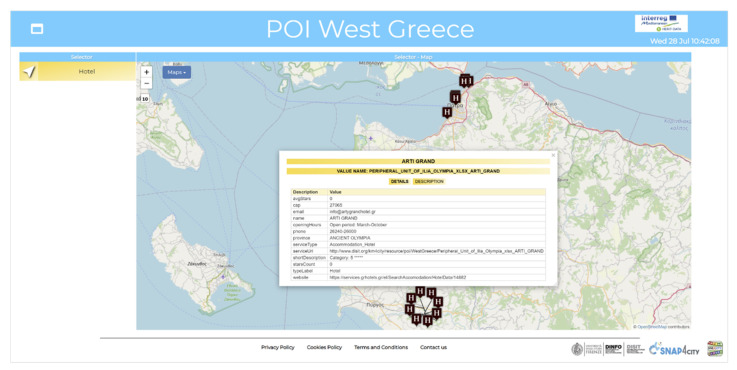
Dashboards representing data collected by exploiting the data ingestion tool according to the Φ*_poi_* formal model: POIs (hotels) in western Greece map, showing static attributes.

In Figure 8 a dashboard is depicted that represents some POIs (hotels) in western Greece by exploiting the multi-data map widget. These data are collected and modeled as POIs from ${𝕗}_{poi}$ Excel files (according to the formal model illustrated in Section “*Formal Model for POIs*”). Static attributes ingested for each POI (e.g., address, phone, email, description, and stars) are shown in the *Details* tab of an informative popup which can be opened by clicking on each map marker. In the description tab of the same popup some descriptive attributes of the formal model, $\Phi_{poi}$ are shown, such as nature, n, and subnature, sn.

## 7. Conclusions

The arrival of the IoT has opened the path for creating a large number of devices with corresponding data models. The proliferation of data models has also been stressed by the concept of digital twins. Thus, a number of entities are pushing in the direction of creating data model standards for interoperability. On the other hand, a strong push on interoperability with legacy systems, including physical and virtual data, would be needed, while thousands of different emerging standard models are competing with the possibility of defining new models at any time. Thus, any solution to claim to be interoperable has to be capable to cope with multiple standards and models, such as the solution proposed. The data collection and interaction with physical and virtual sensors/actuators, POIs, and KPIs is now a reality. Smart cities and Industry 4.0 are the first domains in which these opportunities have been taken.

On the other hand, due to the high heterogeneity of data, data models can be hardly compacted to create standard formats. On the other hand, data regularization is mandatory to establish relationships among them and thus to make them immediately usable by visual analytics tools, APIs, data analytics, and other devices. The solution proposed in this paper addressed the problem by automating the activities of understanding data at the ingestion phase, preparing them for rendering in an integrated manner with any other data of the platform (in terms of data hypercubes, time slices, time series, trajectories, etc.). In reality the solution can analyze data loaded as Excel files, extract models, and automatically generate IoT device models, templating from them the IoT devices and time series data. This allows the easy upload of KPI and historical data, shortening set-up activities whenever needed, namely when the IoT device stream in real time is connected to the platform. The solution can be used for the massive registration of devices and data in Snap4City multitenant architecture respecting the GDPR compliance. Both model and process have been formalized to cope with all possible operators which may be used for data access and visual rendering. The IoT device model proposed is formalized in FIWARE NGSI V2 and provides a data-variable characterization in terms of value type, value unit, and data type. These aspects allow heterogeneous data collected as hypercubes to be aggregated into a set of aligned hypercubes of cubes, on which it is possible to apply a number of operators, such as slices, and drill down to provide ready-to-use views via the Dashboard Wizard.

The solution has been developed in the context of the HERIT-DATA Interreg project with six different pilots in Europe (Florence in Italy, Pont du Gard in France, Mostar in Bosnia–Herzegovina, Dubrovnik in Croatia, Patra in Greece, and Valencia in Spain). They have provided many datasets in Excel files regarding city evolution, people flow, and tourism. Thus, a large number of heterogeneous data coming from several operators has been ingested and aggregated to provide representative dashboards that city operators can exploit for business intelligence purposes. The solution has been developed on top of Snap4City infrastructure and tools [15]; many dashboards related to those city cases are publicly accessible. After the early validation on HERIT-DATA pilots, the solution has been made accessible for the whole community and organizations using the Snap4City.org platform. The present solution covers more than the 95% of the requirements provided by HERIT-DATA partners. Future work is needed to develop new visual views to reach 100% of the expected requirements. In reality the data model covers the whole set of expected features, while visual representations present some limitations and the highest development costs.

## Figures and Tables

**Table 1:** Certain data structures in the IoT domain and their differences.

Category of Data Model	Geolocation	Static Attributes of the Single Category of Elements	Static Attributes of the Single Element	Dynamic Variables/Time Series
IoT mobile device	Dynamic GPS	Yes	Yes	Yes
IoT device	Static GPS	Yes	Yes	Yes
KPI	Dynamic GPS	None	Yes	Yes
POI	Static GPS	Yes	Yes	None

**Table 2:** The most relevant tests performed on the ingested IoT device data and KPIs. The columns represent the features of the different data ingested while the rows stand for the different visual representations that have been expected and validated to be produced just selecting them from database listings via the wizard.

Widgets	Data with Value Type	Data with Value Unit	Linear Data	Time Series	Time Slice	Drill Down on Time	Map Slice
Multi data map	Multiple	Multiple	No sense	No sense	No sense	No sense	Trajectory if mobile
Time trend	Single	Single	No sense	Yes	Yes	Yes	Trajectory if mobile
Multi time series	Multiple	Single	No sense	Yes	Yes	Yes	Trajectory if mobile
Bar/value sets	Multiple	Single	Yes	No sense	No sense	No sense	Multiple positions
Bar series	Multiple, single	Single, multiple	No sense	Yes	Yes	No sense	Multiple positions
Kiviat, spidernet	Multiple	Multiple	Yes	No sense	Yes	No sense	Multiple positions
Calendar	Single	Single	No sense	Yes	Yes	Yes	Trajectory if mobile
Donut	Multiple, single	Single, multiple	No sense	No sense	Yes	No sense	Multiple positions

**Table 3:** Overview of data sources ingested in the Snap4City platform, represented in terms of Dashboard Wizard instances.

Category of Data Model		Total Number of View Instances on Dashboard Wizard	Total Number of Entities	Total Number of Attributes/Variables
Devices	ETL sensor device	103,222	2794	100,428
	IoT device	18,870	2178	16,692
	IoT mobile device	32	9	23
	Data table	1611	228	1383
Models	IoT device model	96	-	-
	IoT mobile device model	2	-	-
	Data table model	18	-	-
	KPI	168	-	-
	POI	557	-	-

## Data Availability

Most of the data reported are publicly accessible on https://www.snap4city.org.
